# Systems Policy Analysis for Antimicrobial Resistance Targeted Action (SPAARTA): A Research Protocol

**DOI:** 10.12688/wellcomeopenres.22923.2

**Published:** 2025-07-30

**Authors:** Raheelah Ahmad, Nina Zhu, Rishabh Jain, Jyoti Joshi, Mirfin Mpundu, Paola Amigo Gutierrez, Alison Holmes, Tillman Weyde, Rifat Atun

**Affiliations:** 1 Department of Infectious Diseases, Imperial College London Faculty of Medicine, London, England, W21NY, UK; 2 Department of Health Services Research Management, City St Georges University of London School of Health & Psychological Sciences, London, England, EC1V0HB, UK; 3 Dow University of Health Sciences, Karachi, Sindh, 74200, Pakistan; 4 SEDRIC, London, NW12BE, UK; 5 International Centre for Antimicrobial Resistance Solutions, Copenhagen, Denmark; 6 REACT Africa, Lusaka, Zambia; 7 School of Science & Technology, City University of London Department of Computer Science, London, England, EC1V0HB, UK; 8 David Price Evans Chair in Global Health and Infectious Diseases, Pharmacology & Therapeutics, University of Liverpool Institute of Systems Molecular and Integrative Biology, Liverpool, England, L78TX, UK; 9 Imperial College Healthcare NHS Trust, London, England, W21NY, UK; 10 Department of Health Policy & Management, Harvard University T H Chan School of Public Health, Boston, Massachusetts, MA 02115, USA

**Keywords:** ‘Antimicrobial resistance policies’, ‘national action plan’, ‘surveillance’, ‘Health systems’

## Abstract

**Background:**

The majority of countries (88%) have an Antimicrobial Resistance (AMR) National Action Plan (NAP V.1.0), but many remain unimplemented, and lack funding for interventions. Intervention selection requires a systematic approach to explain and predict progress. Looking beyond AMR is important to ensure the capture of systemic factors at the country level, which can impede or accelerate success.

**Aim:**

To provide innovative policy analysis to allow country comparison and refine targeted action, while developing and implementing NAPs (V.2.0).

**Methods:**

Mixed-method multi-country case study of policies and implementation strategies to address AMR across One Health. Starting with 17 countries, the sample includes each WHO region and emerging economies.

This investigation of structures, processes, and outcomes has three components:

a. Textual analysis of peer-reviewed literature, policy documents, global, national and state level progress reports, validated by global and in-country experts. An all-language article search conducted for 2000-2024, using broad search terms: ‘Antimicrobial resistance policies’, ‘national action plan’, ‘surveillance’, ‘AMR systems’ supplemented by hand searches. Deductive analysis using multi-disciplinary frameworks including the Expert Consensus for Implementation Research (ERIC).

b. Longitudinal quantitative analysis assessing country contextual determinants and Antimicrobial Use (AMU) and AMR outcomes. Data from global health indicator repositories and international and national AMU and AMR surveillance networks are analysed using econometrics and machine learning approaches.

c. Interactive Tableau dashboard development to display insights from a & b to allow visualisation and comparison of case-country AMR intervention context and components.

**Discussion:**

This protocol provides a systematic, transparent approach for countries to benchmark their own AMR strategies. The interactive dashboard will allow comparisons between country clusters by geography or economy, and enable rapid knowledge mobilisation among strategic and operational stakeholders including policy makers and planners. This protocol facilitates others to perform this structured assessment and nominate their country for the next wave of analysis.

## Plain Language Summary

Antimicrobial Resistance (AMR) is when microbes become resistant to the drugs (antimicrobials) used to treat them. As this poses a growing problem to society, many countries have developed a National Action Plan (NAP) to outline their strategies to address this problem of AMR. There is limited funding to carry forward the plans, and limited knowledge of which strategies are the most effective. We therefore, need a better understanding of why some countries are more successful than others in using antimicrobials only when needed and reducing infections that are non-treatable by antimicrobials. Our study aims to create a standardised approach for evaluating AMR NAPs through policy analysis as well as developing a tool to enable countries to compare performance and promote knowledge sharing. The investigation has three components: a. Text analysis of the existing literature and policies from multiple countries, b. Data analysis of the factors within countries that affect the use of antimicrobials and the development of AMR, c. Developing a tool to visualise and compare country data on AMR interventions.

This will enable the creation of a method for countries to better understand their AMR situation, compare strategies and use resources most effectively.

## Introduction

The majority of countries (88%) have developed a National Action Plan (NAP version 1.0) in response to the launch of the World Health Organization’s Global Action Plan ( WHO, 2015) ( TrACSS, 2024) and many are developing the next NAP versions, while the burden of Antimicrobial Resistance (AMR) ( Murray *et al.*, 2022) remains unabated. While policy formation is important and legitimises a cause, many NAPs remain to be implemented, and fewer (11%) have associated funding for agreed activities ( TrACSS, 2024). Assessing country-level implementation strategies against the compendium of available options can help explain, and potentially predict progress ( Murray *et al.*, 2022; WHO, 2019b). Mechanisms for ensuring that the evolving evidence base is used to refine policies at the national and local levels are not well established and policy planning processes are usually not agile enough to respond to such evidence ( Charani *et al.*, 2021; Charani *et al.*, 2023; WHO, 2021). For most effective policy planning and implementation, we additionally need to learn from previous and concurrent global health challenges including successes, reasons for stagnation, and failures. Looking beyond AMR is important so that we do not re-invent solutions and ensure that we capture systemic factors at the country level, which can impede or accelerate success. This approach is also important due to the co-dependence between AMR and the Sustainable Development Goals (SDGs) ( Jasovský *et al.*, 2016). Examples for learning include but are not limited to: other infectious diseases (such as TB, HIV/AIDS), mental health, and climate change ( Pitchforth *et al.*, 2022). Epidemics and pandemics (Ebola, COVID) are another obvious source of learning ( Ahmad *et al.*, 2021; Pitchforth *et al.*, 2022; Zhu *et al.*, 2021).

Evidence needs to be timely and needs to make sense to technical experts as well as wider decision-makers, and to ensure that the ‘value proposition’ is clear from scientific, economic, political, and sociological perspectives ( Birgand *et al.*, 2022; Greenhalgh *et al.*, 2017). There are a wide range of perspectives which can be used to frame global AMR but at the national level this may need to be reframed in order to mobilise actions ( Khurana *et al.*, 2023).

There is a need to explore innovative approaches to policy development and implementation to address AMR which could be useful and generalisable across countries. Resources and other contextual factors are important to consider, and there may be other ways to cluster countries to enhance comparative learning, aside from high, middle, and low-income groups ( Cocker *et al.*, 2024; Mounier-Jack *et al.*, 2017). There needs to be theoretically sound, multidisciplinary analysis, which looks at process, determinants, and outcomes at country level and where results are validated by global and in-country experts to ensure relevance to context.

The current research addresses priority questions highlighted by the high level United National General Assembly ( UNGA, 2024). Specifically, *What strategies can countries employ to leverage domestic resources effectively and sufficiently to address AMR across sectors, especially where there are competing development priorities? How can we ensure that AMR NAPs are costed, budgeted, and monitored?* *What strategies can be employed to enhance collaboration and coordination across sectors in countries for AMR response? How do we ensure the private sector is engaged and committed? Which countries are likely to work together in tackling AMR burden? Which countries have similar approaches in tackling AMR? What lessons have been learned from the implementation of the Global Action Plan on AMR over the past nine years? And how can the Global Action Plan be further strengthened?* ( United Nations General Assembly (UNGA), 2024).

A recent *Lancet* series on AMR provides key evidence on interventions and investments to inform decision making to achieve sustainable access to effective antibiotics and accelerate progress in addressing AMR, as well as proposing achievable global targets in humans and animals for 2030. There is consensus ( The Lancet, 2024) that the high overall burden of bacterial infection and AMR is a symptom of global health inequities that are not addressable unless the agenda is re-focused on low and middle as well as high-income countries. Robust evidence of impact of preventative approaches including access to safe drinking water, effective sanitation, vaccination, and infection and prevention control in healthcare facilities shows that these interventions could prevent more than 750,000 deaths associated with bacterial AMR each year in lower middle income countries (LMICs), with additional health and societal benefits ( Patel *et al.*, 2023; The Lancet, 2024).

From a health systems perspective, AMR-specific and AMR-sensitive activities need to be assessed to ensure that resources are effectively deployed and that monitoring of unintended consequences is in place. Many existing wider public health interventions have huge potential to reduce the spread of AMR if they are more broadly implemented. The rising resistance to first-line treatments poses a major risk to the success of HIV, TB and malaria programmes, so preventing AMR is already key to wider health outcomes ( Jasovský *et al.*, 2016; Majumder *et al.*, 2020). There are lessons to be learned, and scope to build on the practical experiences of these programmes. Integrating approaches with existing programmes may result in efficiencies and more sustainable systems ( WHO, 2019a).

The **aim** of this research is to provide innovative, systematic and comprehensive policy analysis to allow countries to compare, refine, and operationalise targeted action to address AMR, while developing and implementing AMR NAPs (version 2.0). Looking beyond AMR is important to ensure capture of systemic factors at the country level, which can impede or accelerate success.

This protocol follows the quality criteria for methods set by the Integrated Quality Criteria for Review of Multiple Study Designs (ICROMS), see Data Availability section ( Zingg *et al.*, 2016).

## Methods

This mixed-method multi-country case study will provide a systematic, comprehensive, and comparable situation analysis of policies and implementation strategies employed to address AMR at country level across One Health (OH).

**Figure 1.**
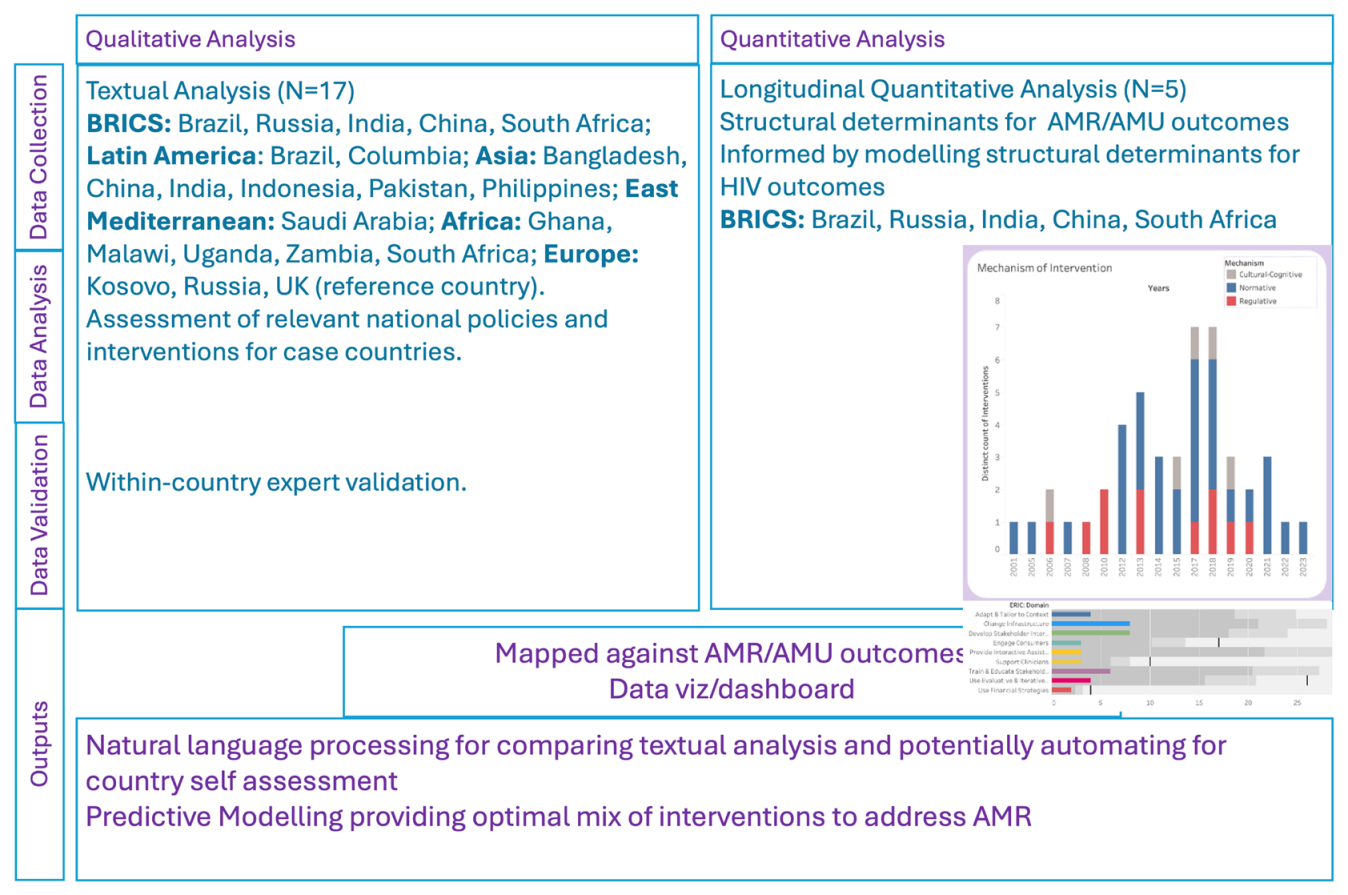
Overview of study methodology.

This investigation of relevant structures, processes, and outcomes at country level, has three components ( Figure 1) including a. Textual qualitative analysis to identify and code interventions for addressing AMR b. Longitudinal quantitative analysis of contextual determinants and outcomes; specifically, antimicrobial use (AMU) and AMR burden c. Interactive dashboard development to allow visualisation and comparison of context and components of AMR interventions in the case countries.

### a. Textual analysis

A textual qualitative approach is used to enable an in-depth appraisal of all policy and intervention types. Deductive analysis is used to ensure a systematic approach to coding. Textual analysis of peer-reviewed literature (Pubmed, Medline, Embase, Global Health), policy documents, global, national and state level progress reports with validation by global and in-country experts. All-language article search conducted for years 2000–2024, using search terms: ‘Antimicrobial resistance policies’, ‘national action plan’, ‘surveillance’, ‘AMR systems’, ‘drug resistant infections’, ‘antibiotic resistance’, ‘infection, prevention and control’, ‘antimicrobial stewardship’, ‘treatment guidelines’, ‘one health’, ‘animal health’, ‘zoonotic disease’, ‘livestock’, ‘agriculture’, ‘environmental health’. Deductive analysis using multi-disciplinary framework including the Expert Consensus for Implementation Research (ERIC).

***Sampling.*** Purposive sampling starting with 17 countries, to represent each of the WHO world regions and emerging economies. The countries selected for the case studies (shown in country groupings) are **BRICS:** Brazil, Russia, India, China, South Africa; **Latin America**: Brazil, Columbia; **Asia:** Bangladesh, China, India, Indonesia, Pakistan, Philippines; **East Mediterranean:** Saudi Arabia; **Africa:** Ghana, Malawi, Uganda, Zambia, South Africa; **Europe:** Kosovo, Russia-U.K.

***Data sources.*** To map policy interventions for the period 2000–2024, we purposefully sampled secondary data sources from peer-reviewed and grey literature. Peer-reviewed articles in all languages are identified from the following databases: Pubmed, Medline, Embase, Global Health. Grey literature including policy documents, global, national and state level progress reports, guidelines, and legislation are sourced using search terms and hand search from: websites of case-country’s health bodies/agencies, and global pan-national websites.

The search terms used are: ‘Antimicrobial resistance policies’, ‘national action plan’, ‘surveillance’, ‘AMR systems’, ‘drug resistant infections’, ‘antibiotic resistance’, ‘infection, prevention and control’, ‘antimicrobial stewardship’, ‘treatment guidelines’, ‘one health’, ‘animal health’, ‘zoonotic disease’, ‘livestock’, ‘agriculture’, ‘environmental health’. Input was also sought from the global expert panel to identify any further within-country or global data sources and documents.

***Data Extraction & Analysis.*** A deductive approach is being used with a range of multi-disciplinary frameworks to extract and code textual data. First the documentary sources are used to extract all interventions which address AMR, AMU, and Infection, Prevention & Control (IPC), and mapped to a timeline for each country. Interventions include all policies, regulations, recommendations, guidelines, plans, monitoring surveillance, campaigns, and activities.

Each intervention is then coded ( Table 1) according to level of implementation (macro, meso, micro), maturity of implementation (developed, implemented, and evaluated), the Expert Consensus for Implementation Research (ERIC) Framework, the PESTELI (Political, Economic, Sociological, Technological, Environmental, Legislative, Industry) Framework, determinants of implementation (barriers, facilitators), setting (secondary, tertiary, specialist care, community and primary care, social care), target/audience (organisations, healthcare professionals and professional groups, patients/patient groups, general public), theme (AMR & AMU surveillance, Antimicrobial Stewardship (AMS), public education and awareness campaign, technology, and Research and Development (R&D), health industry and workforce) pathogen (fungi, bacteria, virus), elements to drive organisational change (Regulative: laws, policies, and contracts, Normative: work norms, habits, cultural-cognitive: beliefs, values). 

**Table 1.** Coding framework for deductive analysis.

	Dimension	Codes
A	ERIC strategy	• Adapt and tailor to context • Change infrastructure • Develop stakeholder interrelationships • Engage consumers • Provide interactive assistance • Support clinicians • Train and educate stakeholders • Use evaluative and iterative strategies • Utilize financial strategies
B1	Level of Implementation	• Macro (international, regional, national) • Meso (organisational) • Micro (individual)
B2	Maturity of implementation	• Developed • Implemented • Evaluated • Evaluation methods: indicators to measure uptake and effectiveness; frequency of review and update
B3	Determinants of implementation	• Barriers • Facilitators
C	Setting	• Secondary, tertiary, and specialist care • Community and primary care • Social care
D	Target / audience	• Organisations • Healthcare professionals and professional groups • Patients / patient groups • General public
E	Theme	• AMR and AMU surveillance • Antimicrobial stewardship (AMS) • Public education and awareness campaign • Technology and R&D • Health industry and workforce • Infection, prevention and control (IPC)
F	Pathogen	Fungi, Bacteria, Virus
G	PESTELI domain	Political, Economic, Social, Technological, Environmental, Legal, Industry
H	Elements to drive organisational change (optional)	• Regulative: laws, policies, and contracts • Normative: work norms, habits • Cultural-cognitive: beliefs, values

The ERIC framework is a set of 73 discrete strategies for implementation, organised within 9 broader domains (adapt and tailor to context, change infrastructure, develop stakeholder interrelationships, engage consumers, provide interactive assistance, support clinicians, train and educate stakeholders, use evaluative and iterative strategies, utilise financial strategies), which can help with planning implementation and evaluating what has been done, in a structured way ( Powell *et al.*, 2015) The PESTELI framework draws attention to the following domains: Political factors, Economic influences, Sociological trends, Technological innovations, Environmental factors, Legislative requirements, Industry analysis to assess the macro-enviornment ( Ahmad *et al.*, 2019).

Coding is carried out in Excel by selecting sub-domains (Yes/No), if articulated in the intervention description. Coding is conducted independently and systematically by three researchers, with 10% of the sample looked at by all three and any disagreements are solved by group discussion and consensus. Final validation by a fourth reviewer, the within-country expert, who validates coding of 30% of the identified interventions (adapted from ( Mizuno *et al.*, 2018)).

The peer-reviewed articles are additionally coded for barriers and enablers to addressing AMR.

An inductive thematic analysis is used ( Thomas & Harden, 2008), informed by theoretical approaches from the field of health systems strengthening and from institutional theory ( Kyratsis *et al.*, 2019).

### b. Longitudinal analysis

Longitudinal analysis assessing potential impact of contextual structural determinants and AMR interventions on the two dependent variables: AMU and AMR. Data from repositories of global health indicators and international and national AMU and AMR surveillance networks are analysed using econometrics and machine learning approaches.

***Sampling.*** The BRICS countries (Brazil, Russia, India, China, South Africa) are selected as they collectively encompass 45% of the global population and 33% of the global Gross Domestic Product (GDP) ( Henley & Partners, 2024). Mitigating productivity losses due to AMR morbidity and mortality, in these five emerging world economies could allow them to reach their full economic potential with substantial global impact. Each have fully developed AMR NAPs but with varying levels of implementation. Analysing these countries with diverse structural, cultural, and health system contexts provides a means for benchmarking “within-region” countries as well as the future key economies (Mexico, Indonesia, Nigeria, Turkey (MINT)) ( Coque *et al.*, 2023; O'Neill, 2016).

***Data sources.*** We identified a collection of candidate-independent variables for each of the BRICS countries from multiple global health data repositories, including the Global Health Observatory (GHO), World Bank Open Data, and the Organisation for Economic Co-operation and Development (OECD) data. These global health databases ( Table 2) collate evidence and statistics by country, to describe public health contexts and track country progress towards SDGs, which provide the most comprehensive collection of social determinants of health.

**Table 2.** Data sources for AMR and AMU

Country	AMR	AMU
International	WHO Glass Report 2014, 2021, 2022 One Health Trust ResistanceMap	WHO GLASS-Implementation Report 2016–17, 2017–18, 2020 One Health Trust ResistanceMap
Brazil	ReLAVRA: 2011–2014 (Spanish) report ReLAVRA: 2014–2016 report	
Russia	AMRmap national dashboard: 2011–2021 https://amrmap.net	As AMR
India	NCDC NARS-Net report: 2017–2023 report	
China	CHINET: 2011–2023 http://www.chinets.com CARSS: 2011–2023 http://www.carss.cn/	NHC中国抗菌药物临床应用管理和细菌耐药现状 2016 (2010–2015 data): report 2018 (2011–2017 data): report 2021, 2022 report available in hard copy
South Africa	NICD dashboard: 2012–2023: https://mstrweb.nicd.ac.za DoH: 2021 report	

***Data Extraction & Analysis.*** Regional and national surveillance systems and dashboards were searched to develop a panel dataset of AMR levels and AMU for each of the BRICS countries for a minimum of 20 years. We measured country-level AMU using total Defined Daily Dosage (DDD) of antibiotics dispensed to the human population. We considered how variation in data sources might influence the AMU data, so the data from monitoring hospital and community prescribing and dispensing, versus data from monitoring retailers, or import/export of antimicrobials. We are likely to under estimate AMU at global level. Each country’s AMR burden was measured using the reported percentage of resistant isolates for the critical and high priority therapy-pathogen combinations defined by the WHO ( WHO, 2024), including enterobacterales resistant to carbapenems (meropenem, ertapenem, imipenem, and in rare cases, doripenem, panipenem/betamipron, biapenem, and tebipenem), enterobacterales resistant to 3 ^rd^ generation cephalosporins (cefotaxime, ceftazidime, and ceftriaxone), and methicillin-resistant *Staphylococcus aureus* (MRSA). The worst-case scenario was taken if multiple antibiotic agents were tested for one pathogen (i.e., enterobacterales isolates 12% resistant to imipenem, 10% resistant to ertapenem, a resistance level of 12% is used) We are likely to have a skewed picture or only partial capture of the true burden of AMR since most countries report AMR data from secondary or tertiary care levels.

The candidate contextual-independent variables extracted from the global health data repositories were reviewed by the study advisory group panel of experts to generate consensus on which categories of these variables should be the initial focus of the longitudinal analysis, considering prior knowledge of potential impact of these variables on AMR. For instance, variables measuring the process and outcomes of other public health interventions considered less relevant to AMR (e.g., Resources for Substance Use Disorders), were excluded from the analysis. The variables included for analysis are organised under three categories: health system financing, health technologies, and health workforce. The variables within each of the included category are reviewed to identify duplication and the measures of the same objects with different units (e.g., crude number vs density), and the variables included are ones that are adjusted for country variation (i.e., age-standardised percentage is preferred over crude numbers) and with a minimum of 10 years of data. Multicollinearity between independent variables will be quantified using Pearson correlation coefficients ( Sedgwick, 2012), principal component analysis (PCA), and Variance Inflation Factor (VIF) ( Kherif & Latypova, 2020).

To assess the potential country-level impact from the independent variables and AMR interventions on the two dependent variables (AMR and AMU), we employed both econometric models and Machine Learning (ML) causal inference to maximise the validity of this analysis. We developed multivariate beta regression models ( Ferrari & Cribari-Neto, 2004) for each pathogen-therapy combination, estimating parameters by maximum likelihood. Extreme Bounds Analysis (EBA) was performed to assess the robustness of independent variables and Bayesian Model Averaging (BMA) to address model uncertainty. EBA incorporates prior knowledge and attempts to determine the most extreme possible estimates for a fixed subset of coefficients ( Leamer, 2010). It is a type of sensitivity analysis that provides upper and lower limits for the outcome variable for any possible set of determinants so that the determinants robustly associated with the outcomes across many possible scenarios can be identified. It is particularly useful when dealing with a large number of possible explanatory variables and enables testing for whether minor changes in the examined determinants can significantly alter the outcome variables. If the association between a determinant and the outcomes does not vary much across regressions, it is considered robust. EBA supports empirical research by demonstrating the inferential sturdiness of hypotheses (i.e. the robustness of the inclusion or exclusion of a variety of plausible explanation of an observation) ( Hauck *et al.*, 2016). BMA is a statistical technique that addresses model uncertainty by averaging over a set of plausible models rather than selecting a single "best" model ( Wasserman, 2000). BMA assigns probabilities to each plausible model using Bayes' theorem by averaging the parameter estimates from each model weighted by their posterior probabilities. The results from the EBA and BMA analysis will provide insights in how reliable the beta regression model is in capturing the association between the AMU and AMR outcomes and the contextual independent variables. Bayesian networks, a machine learning based tool, are increasingly used for causal inference, decision support and understanding complex probabilistic relationships between variables ( Teles *et al.*, 2014) ( Pérez *et al.*, 2021). A Bayesian network consists of nodes representing variables (e.g. percentage of enterobacterales isolates resistant to carbapenems) and directed edges (e.g. connection between percentage of enterobacterales isolates resistant to carbapenems and population mobility/international travel) representing probabilistic dependencies between variables that contribute to the development and emergence of AMR. Each node has a conditional probability distribution that quantifies the effects of the parent nodes on the node. By analysing the network (the directed acyclic graph (DAG)), key risk factors and pathways leading to the changes in AMU and AMR can be identified, and predicted given certain conditions or interventions. To guide decision makers in terms of policy mix, we use the two modelling approaches to predict how the identified interventions (single or in combination, and sensitivity analysis based on varied level of implementation from partial to complete) would affect a country’s AMU and AMR level, in combination with the contextual independent determinants. Anticipating that the AMR data would be generally less comprehensive and consistent across countries, we used HIV/AIDS prevalence as the outcome measure for model training and development in parallel with AMR and AMU data collection.

This study will be conducted in phases, in phase 1, the study will assess interventions and determinants in the human health sector and their potential impact on AMU and AMR outcomes in BRICS countries. Phase 2 will expand the quantitative assessment to include the environmental and animal health sectors in a different set of case countries.

### c. Interactive dashboard development

Interactive Dynamic Dashboard development to display insights from A. & B., using Tableau to allow visualisation and comparison of context and components of AMR interventions in the case countries.

Using data visualisation, the aim is to present the output of the analysis of interventions so that geographically or economically close countries can compare and reflect on alternative approaches and actionable insights ( Kim & Huang, 2021). The data and results visualised include country demographic profiles, socioeconomic status, AMU and AMR levels, the implementation of AMU and AMR surveillance, participation in surveillance networks, as well as AMR interventions implemented in human health and across One Health.

The benefits of interactive dashboards include the ability to display aggregated data and complex visual analytics embedded in a user-friendly platform ( Thoma *et al.*, 2020). The intended users (policy makers, planners, and commissioners of funding) can navigate through curated visualisations, filter specific details, make comparisons, and uncover insights that are useful for decision-making.

In developing the dashboard, through stakeholder engagement, design-based approach will be adopted to ensure a user-need-informed design.

The dashboard development follows a participatory iterative process including: 1) Desk review of existing AMR policy dashboards to understand structure, functionality, define data sources, data preparation, analysis approach definition, etc. 2) Roundtable discussions with the study advisory group members and stakeholders from the Wellcome Trust and Fleming Fund to understand user needs and applicability in LMIC, HIC, utility within their respective programs of work, 3) Design, construction, and validation with project team and end users, 4) Launch and dissemination through SEDRIC and its peripheral network. Detailed steps in Dashboard Development:

**Platform Selection:** Desk-review of existing AMR policy dashboards to understand structure, functionality, define data sources, data preparation, analysis approach definition, etc. e.g., UKHSA COVID-19 data dashboard, IHME Global Burden of Disease Dashboard, WHO GLASS, CDC AR&PSP, EARS-Net, AMRSNET, AURA, PAHO/WHO Regional AMR, WPRO AMR surveillance, etc.

Multiple data visualisation software were considered (Power BI, Tableau, Data-flo, Pathogen Watch, Echarts, Vizhub) against five main criteria: publicly accessible, flexibility for broader applications outside domain, pre-built functionalities, drag and-drop interfaces, and extent of domain-specific knowledge required, and ease of local adoption.

The generic framework tableau was selected through a pragmatic approach to create the dashboard.

**Data Sourcing:** Outcomes from the deductive structured qualitative analysis which are categorical data variables including those generated from the ERIC coding, geographic/countries, and years. From the quantitative longitudinal analysis, the AMR and AMU indicators at country level and the underlying contextual determinants.

**Data Processing:** Normalisation of data in a structured and readable format for the platform.

**Data Analysis:** Visual analytics composed by temporal analysis, exploratory data analysis, comparative analysis, and geographical visualisation. Temporal analysis aims to discover the trends that can be derived from the data. Exploratory data analysis is focused on analysing the distribution and relation between relevant features (e.g. yearly distribution, focus of intervention). Comparative analysis seek to highlight multiple variable differences effectively. Geographical visualisation facilitates a simple representation of the data to identify and explore trends geographically.

**Testing and Validation:** Presentation and agreement with stakeholders on quality assurance, layout and colour, visual balance, filters, intuitive navigation, and interactive elements ( Bach *et al.*, 2022).

## Discussion

This manuscript provides a detailed protocol including rationale for the research and methods for data collection and analysis. This work is conducted by an international multi-disciplinary team. The advisory team provide input periodically (every 3 months) to ensure relevance of the work.

While the Global Research on Antimicrobial Resistance (GRAM) study has provided much needed quantification of AMR burden and a renewed call to action, comprehensive insight of interventions in different country contexts is needed to inform decision making and enable evaluation ( Murray *et al.*, 2022; Naghavi *et al.*, 2024).

The research approach is timely given the recent Lancet commission ( The Lancet, 2024), and the United Nations General Assembly (UNGA) high level meeting ( United Nations General Assembly, 2024) adding to the suite of tools available to follow through with recommendations from these activities ( Wellcome, 2024).

Major strengths of the study include use of the ERIC framework to sensitise decision makers to the full compendium of options that are available to address AMR. Additionally, the outputs on the dashboards will include: (1) Display of time analysis visualisations to present the yearly distribution of interventions, highlighting trends and changes over time; (2) Display of the distribution and allocation of ERIC strategies across different countries and years, allowing for detailed comparison and analysis of implementation strategies; (3) Visualising of multiple dimensions that describe characteristics of the interventions and their implementation process, which helps understanding of the diverse landscape of AMR efforts; (4) Enablement of comparisons between different countries, different periods of time, or different parameters of implementation, allowing users alternatives to analyse and evaluate the impact of different strategies.

**Study Limitations:** There are gaps in AMR data and structural determinants at country level for any quantitative study because of inconsistency in AMR data collection across different regions and healthcare settings since many countries lack standardized protocols for collecting and reporting AMR data. The sample does not include countries of conflict which have unique challenges and required interventions ( Pallett *et al.*, 2023; Rizk *et al.*, 2021). These countries would provide a different ‘grouping’ or filter in the data visualisation dashboard as the work progresses further. Limitations of the study also include the constraints of customisation options in Tableau but this is balanced by expense to maintain and expand the dashboard.

The outputs from this study will be shared early with the WHO, The Fleming Initiative, and other organisations that have a strong convening power which can help build local consensus, promoting development and uptake of recommendations. These will include The Fleming Fund as it progresses through Phase 2 of implementation, and the Second Trinity Challenge, both aimed at reducing the impact of AMR with data-driven approaches focusing on low- and middle-income communities.

Overall, this protocol provides a systematic and transparent approach for countries to benchmark their own strategies to address AMR while understanding context. The interactive dashboard will allow comparisons between country clusters by geography or economy, helping policy makers and planners. The interactive dashboards will enable rapid knowledge mobilisation among strategic and operational stakeholders. This protocol enables others to engage with this structured assessment approach and nominate their country for the next wave of analysis.

By looking across systems and sectors, there may be an emergent value proposition which resonates with national level stakeholders. Effective and efficient policy change might be achieved, if the solutions and arguments presented to solve the problem are credible, relevant, and feasible.

The systematic, comprehensive approaches employed in this analysis can also serve as a template to develop tools for decision-making and health planning to address other public health issues.

## Ethics and consent

Ethical approval and consent were not required.

## Data Availability

No data associated with this article. Repository name: Systems Policy Analysis for Antimicrobial Resistance Targeted Action (SPAARTA): A Research Protocol, https://doi.org/10.6084/m9.figshare.27008017.v1 ( Ahmad, 2024) This project contains the following underlying data: Supplementary 1 - Search Strategy Supplementary 2 - AMU AMR Global Data Availability (Global/Regional Surveillance) Supplementary 3 - National Surveillance Supplementary 4 - Identified data from global/regional surveillance programmes/reports from case countries - year of reporting Supplementary 5 - Expert Consensus for Implementation Research (ERIC) Framework Supplementary 6 - SPAARTA Protocol ICROMS Quality Criteria Data are available under the terms of the Creative Commons Attribution 4.0 International license (CC-BY 4.0).
