# Supplementary material for: Systems Policy Analysis for Antimicrobial Resistance Targeted Action (SPAARTA): A Research Protocol
Source: Wellcome Open Res. 2025 Jul 30;9:700. Originally published 2024 Dec 2. [Version 2] doi: 10.12688/wellcomeopenres.22923.2 (PMC12332476; doi:10.12688/wellcomeopenres.22923.2)
Supplement: Peer review history

# Reviewer report: Reviewer response for version 2

**DOI:** 10.21956/wellcomeopenres.27049.r127912

**Published:** 2025-08-07

**Authors:** Anand Balachandran, Zlatina Dobreva

**Affiliations:** 1 World Health Organisation, Geneva, Switzerland; 2 AMR Surveillance, Prevention and Control Department, World Health Organization, Geneva, Geneva, Switzerland

The revisions address many of the comments raised in Version .1.

Is the study design appropriate for the research question?

Partly

Is the rationale for, and objectives of, the study clearly described?

Partly

Are sufficient details of the methods provided to allow replication by others?

Partly

Are the datasets clearly presented in a useable and accessible format?

Partly

Reviewer Expertise:

Implementation and assessment of national action plans on AMR; Integration of AMR interventions into PHC; People-centred approach to address AMR in human health; Monitoring and evaluation of AMR national action plans; Gender, equity and disability inclusion dimensions of AMR.

We confirm that we have read this submission and believe that we have an appropriate level of expertise to confirm that it is of an acceptable scientific standard.

# Reviewer report: Reviewer response for version 1

**DOI:** 10.21956/wellcomeopenres.25240.r119600

**Published:** 2025-04-01

**Authors:** Anand Balachandran, Zlatina Dobreva

**Affiliations:** 1 World Health Organisation, Geneva, Switzerland; 2 AMR Surveillance, Prevention and Control Department, World Health Organization, Geneva, Geneva, Switzerland

**Background/ Context:**

- The authors might wish to consider de-linking the lack of funding for interventions to the need for more systematic approach to select the most appropriate interventions that can predict progress **. **The identification of effective interventions to address AMU and AMR does not necessarily mean that it leads to funding for those activities – especially in the context of limited national budgets for public health, decline in ODA, and impact of geopolitical instability.
- The authors might wish to update their figures based on TrACSS 2024 data. 88% of WHO Member States ( 170/ 194)  reported having developed a national action plan on AMR. In addition, 64% of WHO Member States are implementing their national action plans on AMR – in an ad-hoc manner. Data portal: www.new.amrcountryprogress.org
- The authors could consider initially reviewing the existing “packages of effective interventions” to address AMR developed by various international bodies based on existing evidence, estimations, modelling and expert opinions from countries, regions and at the global level. Some of the packages, that include cost effectiveness considerations, return on investment, technical feasibility etc.. are noted below.
- The authors could review and reflect upon many “best buy” packages of interventions proposed by the OECD – both for public health, and for One Health (OECD (2018), *Stemming the Superbug Tide: Just A Few Dollars More*, OECD Health Policy Studies, OECD Publishing, Paris, https://doi.org/10.1787/9789264307599-en; OECD (2023), *Embracing a One Health Framework to Fight Antimicrobial Resistance*, OECD Health Policy Studies, OECD Publishing, Paris, https://doi.org/10.1787/ce44c755-en)
- The authors could also review the package of 20 interventions proposed by the World Bank recently - Rupasinghe, N., C. Machalaba, T. Muthee, and A. Mazimba. Stopping the Grand Pandemic: A Framework for Action. Addressing Antimicrobial Resistance through World Bank Operations. Washington, DC: World Bank; 2024. License: CC BY 3.0 IGO. https://hdl.handle.net/10986/41533

- The authors could also review the WHO people-centred approach to address AMR in human health and core package of interventions to support national action plans. The core focus is on addressing inequities, was endorsed by the World Health Assembly in 2024, highlights the interdependence of interventions, highlights the need to address critical broader health system gaps, and barriers faced by people and vulnerable populations, and provides guidance for specific actions at each levels of the health system.

https://iris.who.int/bitstream/handle/10665/373458/9789240082496-eng.pdf

- The authors also may wish to review the “Smart Choice Process” developed by the AMR Policy Accelerator at York University, Canada that is currently being piloted in many countries in Africa to identify effective package of interventions across the One Health sectors.

**Study Design:**

- It will be useful to clarify if this research is purely for human health or as stated in the methods section, to address AMR at country level across One Health.
- For the textual analysis, it will be critical to expand the search terms. It  could include different ways of referring to AMR such as: “drug resistant infections”, “anti-bacterial/anti-fungal/anti-infective resistance”, “antibiotic resistance” and their variations.
- The search terms are also missing important interventions such as “stewardship”,  “national treatment guidelines” “immunization”, “infection prevention and control” “bacteriology laboratory systems”
- If the study is applying a One Health approach then consider including search terms that would cover interventions in other sectors related to animal health, agriculture, the environment. This is missing at this point.
- The list of 17 countries seem very ambitious. While many of the large countries like India have a “National Action Plan”, health interventions are primarily implemented by the States, and so identifying interventions at the national level might not be helpful or drive actions in States.  It might be useful to identify a smaller group of countries whose NAP on AMR is expiring or has expired, and are currently at the stage of conducting an assessment of their implementation.  The proposed research design could then help provide valuable evidence-based policy guidance to countries to help develop new NAPs and cost the most essential interventions as part of developing an operational plan.
- In many of the selected countries conducting a literature search only to identify historically AMR interventions and the level of their implementation in that country will not be enough because the general literature is normally biased towards the Global North and not all interventions will be well documented.
- Having the preferred intervention in the country’ NAP on AMR in turn is not a guarantee that it was implemented in practice and would not give an indication of the extent of its implementation. Pairing this with i) quantitative data from TrACSS and national/regional monitoring reports and with ii) national key informant interviews (beyond just one national expert especially in the case of countries of the size and administrative arrangement like China and Brazil) will be key.
- The authors could clarify how the deductive analysis of AMR interventions will be converted to a quantitative variable that can be used in the longitudinal modelling analysis. Will there be an overall score assigned for the level of implementation of a given intervention in a given year for a given country?
- It would be beneficial to include “Infection Prevention and control”, “ Diagnosis and Bacteriology / Mycology laboratory systems” under Theme “E” in the coding framework for deductive analysis.
- Will Gender and other social stratifiers that impact equitable access be included in the determinants of implementation.
- The authors can clarify how data from quantitative sources will be incorporated at the coding stage for each intervention.
- A major challenge for the longitudinal analysis of the impact of AMR interventions and independent variables on the two AMR outcomes will be the quality of the AMR and AMU data, and in particular changes in the quality of the data over the study period for a given country.
   
  - For example, the latest WHO GLASS report found that percentage of AMR infections was lower in countries that have high testing coverage (number of bacteriologically confirmed infections per million population) within the top 75 ^th^ percentile. If there are significant changes in testing coverage or other factors affecting the quality of the data longitudinally in each of the countries then that can introduce bias in the effect estimates. The authors could consider observing the patterns of AMR/AMU data over time and if there are significant unexpected changes year on year, which would warrant including additional independent variable in the model to control for data quality.
- Another consideration for the Longitudinal analysis would be for the authors to identify a different indicator rather than HIV/AIDS prevalence for each country as an alternative independent variable. Significant systems have already been established for HIV/AIDs in many countries based on single disease financing mechanisms, and this might not be comparable for a complex phenomenon like AMR.
- The use of both Extreme Bounds Analysis (EBA) and ML-based the Bayesian networks as the two modelling approaches would be very beneficial to guide decision-makers at national, as well as regional and global levels. This, at the core, is a very innovative element of the protocol and in comparison to some other existing prioritization processes. The paper would benefit from additional details on the use of the two modelling approaches, including some examples and sample visualization.
- For the Visualization dashboard, the authors may wish to review the new TrACSS website that includes data comparison across countries, regions, across income levels, has global benchmarks for various indicators (across all One health Sectors), as well as a WHO AMR M&E portal (to be ready in April 2025) that will include the human health indicators as recommended in the Global Action plan M&E Framework and recommended indicators.

Is the study design appropriate for the research question?

Partly

Is the rationale for, and objectives of, the study clearly described?

Partly

Are sufficient details of the methods provided to allow replication by others?

Partly

Are the datasets clearly presented in a useable and accessible format?

Partly

Reviewer Expertise:

Implementation and assessment of national action plans on AMR; Integration of AMR interventions into PHC; People-centred approach to address AMR in human health; Monitoring and evaluation of AMR national action plans; Gender, equity and disability inclusion dimensions of AMR.

We confirm that we have read this submission and believe that we have an appropriate level of expertise to confirm that it is of an acceptable scientific standard, however we have significant reservations, as outlined above.

## References

[1] Data portal. Reference source

[2] : Use of human predictive patch test (HPPT) data for the classification of skin sensitization hazard and potency. *Arch Toxicol* .2024;98(5) : 10.1007/s00204-023-03656-4 1253-1269 10.1007/s00204-023-03656-4 38483583 PMC10965744

[3] : Embracing a One Health Framework to Fight Antimicrobial Resistance. 10.1787/ce44c755-en 10.1787/ce44c755-en

[4] *World Bank Group* . Reference source

[5] People-centred approach to addressing antimicrobial resistance in human health. Reference source

# Reviewer report: Reviewer response for version 1

**DOI:** 10.21956/wellcomeopenres.25240.r120264

**Published:** 2025-03-26

**Authors:** Olga Perovic

**Affiliations:** 1 Centre for HAI, AMR and mycosis, University of Witwatersrand, Johannesburg, South Africa

I wish to thank Wellcome for asking me to review this SPAARTA protocol. Overall, it is well described innovative approach to NAPs analysis. Mixed method included textual analysis, longitudinal quantitative analysis, and interactive dashboard to allow visualization of deductive analysis, econometrics and machine learning. LMICs have additional issue and that is a severe lack of funding for AMR program. Also, majority of countries rely on external funding for most of their programs and sustainability of them is questionable. 

 The development of interactive dashboards is of a great value to create Geo Map for AMR and possibility to monitor development of resistance. 

 It is very useful to promote standardized way of consensus approach in development of AMR data repository.

Is the study design appropriate for the research question?

Yes

Is the rationale for, and objectives of, the study clearly described?

Yes

Are sufficient details of the methods provided to allow replication by others?

Yes

Are the datasets clearly presented in a useable and accessible format?

Yes

Reviewer Expertise:

Clinical microbiology, public health, diagnostic stewardship, AMR surveillance, infectious diseases.

I confirm that I have read this submission and believe that I have an appropriate level of expertise to confirm that it is of an acceptable scientific standard.
